# A step toward defining optimal liposomal amphotericin B treatment duration in pulmonary mucormycosis: results from emulated trials

**DOI:** 10.1128/aac.01523-25

**Published:** 2026-04-29

**Authors:** Anne Coste, Olivier Paccoud, François Danion, Anne Conrad, Sylvain Poirée, Amandine Le Bourgeois, Claire Defrance, Valérie Letscher-Bru, Florent Morio, Thomas Gastinne, Marie-Elisabeth Bougnoux, Felipe Suarez, Solène Le Gal, Damien Dupont, Florence Ader, Carine Halfon-Domenech, Sophie Ducastelle-Leprêtre, Françoise Botterel, Laurence Millon, Gaelle Guillerm, Séverine Ansart, David Boutoille, Marie-Pierre Ledoux, Jean-Etienne Herbrecht, Christine Robin, Giovanna Melica, Dea Garcia-Hermoso, Olivier Lortholary, Raoul Herbrecht, Raphaël Porcher, Fanny Lanternier

**Affiliations:** 1 Infectious Diseases Department, La Cavale Blanche Hospital, Brest University Hospital 26990 , Brest, France; 2 INSERM LaTIM, UMR 1101, Université de Bretagne Occidentale 27002, Brest, France; 3 Infectious Diseases Department, Necker–Enfants Malades Hospital, AP-HP, Paris, France; 4 Institut Pasteur, Université Paris Cité, National Reference Center for Invasive Mycoses and Antifungals 555089, Paris, France; 5 Infectious Diseases Department, Strasbourg University Hospital, Inserm UMR_S 1109, Immuno-rhumatologie Moléculaire 36604, Strasbourg, France; 6 Infectious Diseases Department, Croix Rousse Hospital, Hospices Civils de Lyon 26900, Lyon, France; 7 Centre International de Recherche en Infectiologie (CIRI), Inserm U1111, CNRS UMR5308, ENS Lyon, Claude Bernard Lyon 1 University, Lyon, France; 8 Radiology Department, Necker–Enfants Malades Hospital, AP-HP 37072, Paris, France; 9 Clinical Hematology Department, Nantes University Hospital, Nantes, France; 10 Radiology Department, Nantes University Hospital 26922, Nantes, France; 11 Parasitology and Medical Mycology Laboratory, Strasbourg University Hospital, Strasbourg, France; 12 Institut de Parasitologie et Pathologie Tropicale, UR 3073 Pathogens-Host-Arthropods-Vectors Interactions, Strasbourg University 213273, Strasbourg, France; 13 Nantes Université, CHU Nantes, Cibles et Médicaments des Infections et de l'Immunité, UR1155, Nantes, France; 14 Mycology and Parasitology Laboratory, Necker–Enfants Malades Hospital, AP-HP, Paris, France; 15 Unité Biologie et Pathogénicité Fongiques, Institut Pasteur 27058, Paris, France; 16 Hematology Department, Necker Enfants Malades Hospital, AP-HP, Paris, France; 17 Mycology and Parasitology Department, La Cavale Blanche University Hospital, Brest, France; 18 Infections Respiratoires Fongiques, Brest and Angers University, Brest, France; 19 Medical Mycology and Parasitology Department, Institut des Agents Infectieux, Hospices Civils de Lyon, Lyon, France; 20 Institut d’Hématologie et Oncologie Pédiatrique, Hospices Civils de Lyon 26900, Lyon, France; 21 Clinical Hematology Department, Hospices Civils de Lyon, Lyon, France; 22 Mycology and Parasitology Unit, Henri Mondor University Hospital, AP-HP 378967 , Créteil, France; 23 EA DYNAMYC 7380, Université Paris-Est Créteil, Créteil, France; 24 Mycology and Parasitology Laboratory, Besançon University Hospital, Besancon, France; 25 UMR 6249, CNRS Chrono-Environnement, Université de Bourgogne Franche-Comté, Besançon, France; 26 Hematology Department, Morvan Hospital, Brest University Hospital 55162 , Brest, France; 27 Infectious Diseases Department, Nantes University Hospital 26990, Nantes, France; 28 Centre d’Investigation Clinique, INSERM 1413, Nantes University Hospital, Nantes, France; 29 Hematology Department, Strasbourg Cancer Institute, Strasbourg, France; 30 Intensive Care Unit, Strasbourg University Hospital, Strasbourg, France; 31 Hematology Department, Henri Mondor Hospital, AP-HP 36604, Créteil, France; 32 Infectious Diseases Department, Henri Mondor Hospital, AP-HP 378967 , Créteil, France; 33 Translational Mycology Research Group, Mycology Department, Institut Pasteur, Université Paris Cité, National Reference Center for Invasive Mycoses and Antifungals 378967 , Paris, France; 34 Centre d’Epidémiologie Clinique, Hôtel-Dieu Hospital, AP-HP 27058, Paris, France; University of Iowa, Iowa City, Iowa, USA

**Keywords:** emulated trials, isavuconazole, amphotericin B, mucormycosis

## Abstract

Management of pulmonary mucormycosis includes intravenous liposomal amphotericin-B (L-AmB), followed by posaconazole or isavuconazole. We aimed to determine the optimal L-AmB duration before switching to triazole therapy. Using data from a retrospective study in France, we performed an emulated trial evaluating stopping L-AmB after 14 days (short treatment) versus continuing L-AmB (long treatment). The benefit of combination L-AmB therapy was also investigated. A cloning, censoring, and weighting approach was used to account for immortal time and indication biases. Ninety-four cases of pulmonary mucormycosis were included, of which 35% had dissemination. Eighty-nine patients received L-AmB first-line therapy for a median of 25 (IQR: 12–54) days, including 18 patients as combination therapy. L-AmB was discontinued for adverse events in 16/89 patients (18%). Treatment was switched to posaconazole (*n* = 38) or isavuconazole (*n* = 9) in 47 cases and was switched back to L-AmB due to disease progression in 11/47 (23%) cases. Overall, the 180-day mortality was 54%. Diagnosis in the ICU, dyspnea at diagnosis, disseminated disease, and ground-glass opacities on chest CT scan were associated with increased mortality. No difference in 180-day survival was found between patients receiving short or long L-AmB treatment (HR=0.80, 95% CI [0.29–1.99]) and between patients receiving L-AmB alone or in combination (HR=1.14, 95% CI [0.56–2.32]) in emulated trials, although confidence intervals were wide. Switching to triazole therapy after 14 days of L-AmB treatment was as effective as longer durations while reducing L-AmB side effects. Combination therapy did not improve prognosis. These results could inform randomized clinical trials assessing different first-line therapeutic strategies for pulmonary mucormycosis.

## INTRODUCTION

Mucormycosis is the second most common invasive mold infection after aspergillosis in France (1). Pulmonary mucormycosis (PM) is the main form of infection in Europe and predominantly affects severely immunocompromised patients (2, 3). Current treatment guidelines for mucormycosis recommend a combination of first-line antifungals using intravenous liposomal amphotericin B (L-AmB) and surgery, whenever the latter is feasible (4). L-AmB is recommended until stable disease is achieved, but in clinical practice, it is frequently extended more than 4 weeks or until adverse effects occur (5). Posaconazole and isavuconazole have shown promising results as first-line alternatives in non-randomized studies (6, 7), and they are recommended for step-down oral treatment. Results from a prospective cohort study have suggested that a short (<14 days) treatment course with L-AmB may be used successfully in patients with COVID-19-associated rhino-orbital mucormycosis (8). However, severely immunocompromised patients were excluded from this study, and mortality rates were low (2), meaning that these results may therefore not be applicable to patients with PM. It is still unknown if prolonged L-AmB treatment durations before triazole step-down treatment are necessary.

Finally, although combination therapy has demonstrated a survival benefit in animal models (9, 10), data are too limited to support its use in clinical practice.

Randomized controlled trials are challenging to set up because of the relatively low incidence of PM and the heterogeneity of clinical presentations. Despite issues related to positivity violation (i.e., when patients with certain characteristics never receive one of the treatment strategies compared) and residual confounding, target trial emulation is a promising tool to minimize the biases associated with the use of observational data to assess treatment responses, particularly immortal time bias and indication bias (11). We aimed to assess the effect of L-AmB treatment duration on survival and the impact of first-line L-AmB-based combination therapy on survival.

## MATERIALS AND METHODS

### Data sources and definitions

We included patients from a cohort study of patients with PM conducted in six tertiary hospitals across France from January 2008 to January 2019 (12). Cases of mucormycosis with a pulmonary localization, either limited to the lungs or with dissemination, were identified through multiple databases to ensure exhaustivity. All proven and probable cases, according to modified European Organization for Research and Treatment of Cancer/Mycoses Study Group (EORTC-MSG) definitions, were included (*n* = 114). Diabetes mellitus and trauma (in cases with a compatible temporal relationship) were added as host factors. A positive real-time quantitative polymerase chain reaction (qPCR) assay was added as a mycological criterion. For the present study, we excluded 20 patients who were diagnosed with mucormycosis post-mortem. Dissemination was defined as involvement of two or more non-contiguous sites, except for the sinuses. A single “main risk factor” was assigned to each patient in the following order: (i) allogeneic hematopoietic stem cell transplantation (aHSCT); (ii) hematological malignancy (HM); (iii) solid-organ transplantation (SOT); and (iv) other (i.e., non-severely immunosuppressed patients). Neutropenia was defined as a PNN <500/mm^3^ within 1 month before diagnosis. Treatments active against Mucorales included L-AmB, posaconazole, and isavuconazole. Monotherapies that were switched within 3 days to combination therapy were regarded as combination therapies. Response to treatment was assessed according to the EORTC-MSG criteria (13). The cause of death was assessed by two clinicians, based on all available clinical, biological, and radiological data. The cause of death of patients with both a progressive mucormycosis and hematological disease at the time of death was assumed to be mucormycosis related.

### Statistical analysis

Continuous variables are expressed as median (interquartile range) and categorical variables as numbers (percentages). All analyses were performed using R software version 4.0.5 (R Foundation for Statistical Computing).

Two different causal analyses were undertaken to estimate the effect of L-AmB treatment duration, while adjusting for immortal time bias and indication bias (11, 14). We first emulated a trial in which participants with PM still under L-AmB treatment after 14 days would be randomized between stopping or continuing treatment. To accommodate the possibility that eligible individuals might not stop treatment exactly at day 14 but possibly in the next few days, a 7-day grace period was used. The successive steps for trial emulation were as follows: (i) specification of the target trial and eligibility criteria; (ii) cloning participants so that each study participant was allocated to each treatment duration group; (iii) censoring the clones when their actual treatment deviated from their group; (iv) deriving inverse probability weights to account for selection bias due to artificial censoring; and (v) analysis of the data using those weights with additional adjustment for response to treatment at day 14. The primary outcome was survival up to 180 days after randomization, and the between-group comparison was expressed in terms of difference in 45-day survival, the difference in 180-day survival, the difference in 180-day restricted mean survival time (RMST), and the hazard ratio. A similar approach (cloning, censoring, and weighting) was used to emulate a sensitivity analysis trial in which participants would be randomized between receiving ≤28 days or >28 days of L-AmB (Table S1 and S2).

Finally, a trial evaluating the effect of combination therapy on mortality was emulated. To balance groups at baseline, we used inverse probability of treatment weighting. Groups were compared in terms of survival difference, 180-day RMST, and hazard ratio in the weighted sample.

Detailed methods are available in the Supplementary material.

## RESULTS

### Demographic data and underlying diseases

Among 94 cases of PM, 42 (45%) were proven and 52 (55%) were probable. The median age was 58 (42–65) years (Table 1). Hematological malignancy (HM) was the most common main risk factor (*n* = 44, 47%), followed by aHSCT (*n* = 21, 22%) and SOT (*n* = 15, 16%). Forty-one (44%) patients developed mucormycosis while receiving antifungal prophylaxis (breakthrough mucormycosis). Thirty-three (35%) patients had disseminated mucormycosis, among whom 14 (15%) had brain involvement. The median time from first symptom to diagnosis was 13 (7–25) days. Twenty-five (27%) patients were co-infected with *Aspergillus* (as defined by EORTC/MSG criteria).

**TABLE 1:** Patients’ baseline characteristics^*a*^

Patient characteristics	All patients (*n* = 94)	Patients included in the 14-day emulated trial (*n* = 62)	Patients included in the combination therapy emulated trial (*n* = 93)
Age, median (IQR), y	58 (42–65)	56 (41–65)	58 (42–65)
Sex (male)	66 (70)	47 (76)	65 (70)
Main risk factor			
Hematological malignancy	44 (47)	31 (50)	44 (47)
Allogeneic HSCT	21 (22)	14 (23)	21 (23)
Solid organ transplantation	15 (16)	8 (13)	14 (15)
Diabetes mellitus	5 (5)	2 (3)	5 (5)
Other^*b*^	9 (10)	7 (11)	9 (10)
Clinical manifestation			
Fever	73 (78)	52 (84)	73 (79)
Dyspnea	51 (54)	33 (53)	50 (54)
Thoracic pain	22 (23)	17 (27)	22 (24)
Hemoptysis	13 (14)	8 (13)	13 (14)
Neutropenia <500/mm^3^	54 (57)	38 (61)	54 (57)
Disseminated mucormycosis	33 (35)	17 (27)	33 (35)
Mycological identification	78 (83)	51 (82)	77 (83)
*Rhizopus* species	27/78 (35)	14/51 (28)	27/77 (35)
*Rhizomucor* species	20/78 (26)	14/51 (28)	20/77 (26)
*Lichtheimia* species	18/78 (23)	13/51 (26)	18/77 (23)
*Mucor* species	9 /78 (12)	8/51 (16)	9/77 (12)

^*a*^ Data are presented as No. (%) unless otherwise indicated. IQR, interquartile range.

^*b*^ Other: systemic inflammatory disease (*n *= 3), trauma (*n *= 3), chronic granulomatous disease (*n *= 1), extensive burns (*n *= 1), drug reaction with eosinophilia and systemic symptoms syndrome (*n *= 1), and iatrogenic agranulocytosis (*n *= 1).

### Management

Fifty-four patients (57%) received at least one empirical mould-active antifungal prior to formal diagnosis, which was active on Mucorales in only 15/94 cases (16%). Empirical therapy was subsequently changed in 17 cases, resulting in 32/94 patients (34%) with an empirical therapy active on Mucorales (Table 2). Neutropenic patients and patients with a reversed halo sign on CT scan more frequently received Mucorales-active empirical treatment (24/54 = 44% vs 8/40 = 20%, *P* = 0.02 and 13/22 = 59% vs 19/72 = 26%, *P* = 0.01, respectively). Patients under voriconazole prophylaxis or being admitted to the ICU because of mucormycosis symptoms were not more likely to receive Mucorales-active empirical treatment (6/14 = 43% vs 26/80 = 33%, *P* = 0.54, and 15/39 = 39% vs 17/55 = 31%, *P* = 0.51, respectively).

**TABLE 2:** Therapeutic approach^*a*^

	All patients (*n* = 94)
Antifungal therapy active on Mucorales^*b*^	93 (99)
Time to treatment after symptom onset*,* median (IQR), *d*	11 (6–20)
Time to treatment after diagnosis*,* median (IQR), *d*	0 (−3–0)
Initiation before diagnosis	32 (34)
First line including L-AmB	89 (95)
L-AmB dose (mg/kg)**^*c*^**	5 (5.0–6.4)
L-AmB combination therapy	18 (19)
Triazole step-down	47/94 (50)
Intensive care unit admission	44 (47)
Invasive mechanical ventilation	34/44 (77)
Vasopressors	26/44 (59)
Extracorporeal membrane oxygenation	1/44 (2)
Growth factor administration	21 (22)
Excision surgery	14 (15)

^*a*^ Data are presented as No. (%) unless otherwise indicated. IQR, interquartile range.

^*b*^ One patient died while receiving voriconazole therapy.

^*c*^ L-AmB dose was available in 71 patients. L-AmB: liposomal amphotericin B.

Finally, 93 (99%) patients received a systemic antifungal drug active on Mucorales, a median of 11 (7–21) days after symptom onset. Seventy-five patients were initially treated with L-AmB (*n* = 71), posaconazole (*n* = 3), or isavuconazole (*n* = 1) monotherapy. Combination therapy with two antifungals was administered to 18 patients, including 17 patients treated with a combination of L-AmB and posaconazole, and one patient treated with a combination of L-AmB and isavuconazole. First-line therapy included L-AmB at 5 (5–6) mg/kg/day in 89 (95%) patients. Median length of L-AmB treatment was 25 (12–54) days. Sixteen (18%) patients had side effects requiring treatment discontinuation after a median of 22 (9–42) days. In patients receiving immunosuppressive agents or systemic corticosteroids, doses were reduced at diagnosis of mucormycosis in 16/34 (49%) and 16/38 (43%) of patients, respectively. Forty-four (47%) patients were admitted to the ICU a median of 6 (1–18) days after symptom onset, and seven patients developed symptoms associated with mucormycosis while in ICU. Diagnosis was made in ICU in 32 (73%) patients. Length of ICU stay was 18 (9–23) days for surviving patients.

Lung excision surgery was performed in 14 (15%) patients, a median of 21 (2–40) days after diagnosis. Three additional patients underwent emergency surgery for pleural decortication. Only four ICU patients underwent excision surgery, which was performed after ICU admission in one case.

Among patients receiving L-AmB in monotherapy or in combination as first or second-line therapy, a triazole step-down regimen was performed in 47/91 (52%) cases after 33 (15–69) days of L-AmB therapy. Thirty-eight (38/47, 81%) patients received posaconazole, and nine (9/47, 19%) received isavuconazole. Seventeen patients eventually stopped antifungals because of a favorable outcome of mucormycosis after being treated for a median of 14 (8–24) months. No relapse was observed.

### Outcome

The median duration of follow-up in survivors was 32 (19–60) months, with all patients followed more than 180 days. Median survival after first symptom was 87 days. Overall, 180-day mortality was 57% (54/94). Mortality was 66% in HM patients (29/44), 67% in allo-HSCT patients (14/21), 27% in SOT patients (4/15), and 21% in others (3/14) (*P* = 0.03) (Fig. S1). A 180-day mortality up to 93% (13/14) was observed in patients with neurological involvement.

During follow-up, 67/94 (71%) patients died. Death was attributable to Mucorales infection in 47/67 (70%) cases. When considering patients with HM, only 7/39 (18%) deceased patients and 5/5 (100%) surviving patients were in remission from their HM at the end of follow-up. Death from mucormycosis occurred within 23 days after diagnosis in half of cases. The cumulative proportion of deaths according to presumed cause of death during the first 180 days of follow-up is depicted in Fig. S2.

### Prognostic factors

In a multivariable Cox model, diagnosis in the ICU, dyspnea at diagnosis, disseminated mucormycosis, and ground-glass opacities on CT-scan evaluation were associated with death up to day 180 (Table 3). Breakthrough mucormycosis (*n* = 40) and use of growth factors (*n* = 21) were not associated with worse outcomes.

**TABLE 3:** Multivariable analyses using a Cox model for death up to day 180 (hazard stratified on surgical treatment)

	Death up to day 180 HR (95% CI)	
Variable		*P*
Age at diagnosis (per year)	0.99 (0.98 to 1.01)	0.55
Female	1.48 (0.79 to 2.77)	0.22
Diagnosis in the ICU	2.10 (1.05 to 4.18)	0.035
Dyspnea at diagnosis	2.50 (1.31 to 4.76)	0.005
Neutropenia before or at diagnosis	1.91 (0.94 to 3.86)	0.073
Treatment started ≥2 days before diagnosis	1.94 (0.97 to 3.87)	0.061
Disseminated form	2.20 (1.20 to 4.04)	0.011
Pleural effusion on CT scan	1.65 (0.84 to 3.23)	0.15
Ground-glass opacity on CT scan^*a*^		
Main effect	0.14 (0.022 to 0.94)	0.043
× log(Time + 1)	1.99 (1.11 to 3.54)	0.02

^*a*^ Time-dependent effect added because of evidence of non-proportional hazards (*P *= 0.017).

### Emulated trials of L-AmB treatment duration

**Fig 1:**
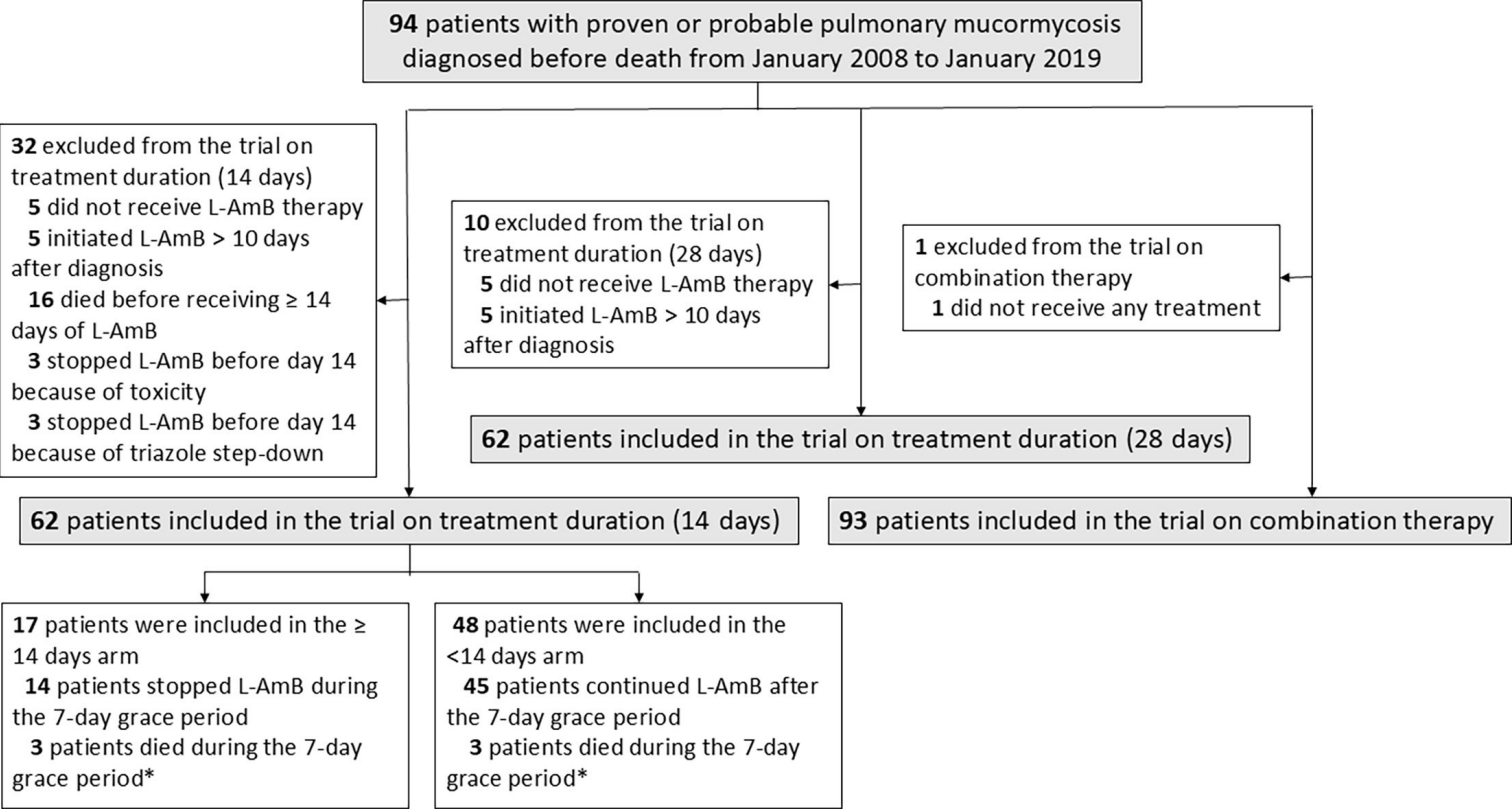
Patients included in the emulated trials on treatment duration (14 days and 28 days) and in the emulated trial on combination therapy.

**Fig 2:**
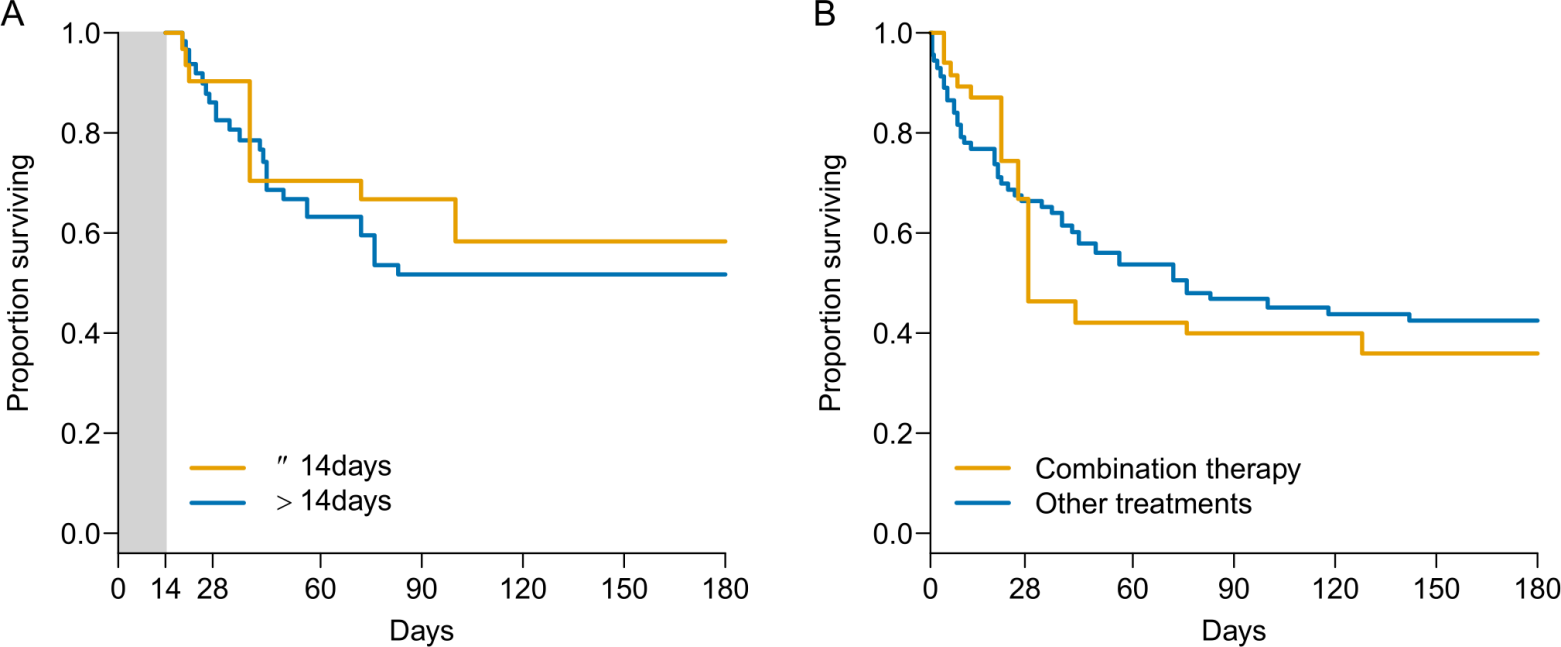
(**A**) Survival according to stopping or continuing L-AmB treatment after 14 days after weighting. (**B**) Comparison of survival in patients receiving an L-AmB combination therapy or L-AmB alone after weighting.

The emulated trial evaluating L-AmB treatment discontinuation at 14 days included 62 patients (Fig. 1). Three patients died under treatment during the grace period and contributed equally to both groups. Balance diagnostics at the end of the grace period, before and after weighting, are presented in Table S3. Given the limited number of participants who discontinued L-AmB at 14 days, inverse probability weighting was unable to balance all characteristics; however, a near-perfect balance was obtained for response to treatment at day 14, a major prognostic factor, and an acceptable balance was achieved for other prognostic factors (e.g., sex, diagnosis in the ICU, dyspnea, and neutropenia). Imbalance on baseline prediction of mortality and treatment started before diagnosis was markedly decreased. We found no evidence of an average benefit of continuing L-AmB treatment beyond 14 days compared to discontinuing treatment at day 14 (HR = 0.80, 95% CI [0.29–1.99]) (Table S4; Fig. 2A). The difference in restricted mean survival time at 180 days was −11 days (95% CI [−46 to 40]).

There was also no evidence of a difference in survival in the emulated trial involving 84 patients, which compared patients receiving ≤28 days of L-AmB versus those receiving >28 days (Fig. 1 and 3; Table S5 and S6).

### Emulated trials on combination therapy

The emulated trial comparing a combination therapy versus monotherapy included 93 patients, of whom 18 received combination therapy (Fig. 1). Balance diagnostics are presented in Table S7. We found no evidence of an average benefit of combination therapy compared to monotherapy (HR = 1.14, 95% CI [0.56–2.32]) (Fig. 2B; Table S8).

### Failure of triazole switch after L-AmB therapy

Triazole therapy was switched back to L-AmB due to disease progression in 11/47 (23%) cases after a median of 20 (14–46) days of triazole therapy, including 7/38 (18%) patients receiving oral suspension (*n* = 6) or posaconazole tablets (*n* = 1) and 4/9 (44%) receiving isavuconazole (*P* = 0·18). Therapeutic drug monitoring was performed in 5/7 patients receiving posaconazole. No significant association was found between failure of triazole therapy and duration of L-AmB therapy before the triazole switch (41 [10–54] days vs 32 [16–73] days, *P* = 0.47), disseminated mucormycosis (*P* = 0.71), or underlying disease (*P* = 0.59). A favorable outcome regarding mucormycosis was eventually observed during follow-up in 7/11 patients requiring a switch back to L-AmB; however, seven patients died within 180 days, including four HM patients with hematological treatment failure.

## DISCUSSION

Trials evaluating L-AmB optimal duration were emulated using data from 94 patients diagnosed with PM. Emulation enabled comparisons while adjusting for immortal time bias, selection bias, indication bias, and response to treatment (14). No difference in mortality was observed between patients stopping L-AmB treatment after 14 days and those continuing L-AmB. This result was supported by a second emulated trial comparing patients receiving L-AmB for ≤28 days or >28 days. To our knowledge, these are the first data evaluating optimal L-AmB treatment durations in patients with PM.

In our experience, patients diagnosed with PM usually receive approximately one month of L-AmB as first-line therapy before switching to oral triazole therapy. Our results suggest that a brief course of liposomal amphotericin B followed by a triazole may be adequate. Shortening the duration of L-AmB treatment may lead to decreased toxicity. Indeed, L-AmB-associated toxicity is also an important matter of concern (15). In our cohort, nearly one of five patients required discontinuation of L-AmB due to toxicity, in most cases after 10 days of therapy. However, our results are limited by the retrospective nature of our study, inherent to the rarity of the disease. Efforts were made to mitigate the risk of immortal time bias, selection bias, and indication bias by applying a target trial emulation framework (16). In particular, we used the clone–censor–weight approach, in which each patient is cloned to each treatment strategy and contributes follow-up time to that strategy up to the time their data deviates from their assigned strategy (11). Moreover, we additionally adjusted for treatment response at day 14 to reduce indication bias. This ensures that the time of eligibility, the assignment of treatment, and start of follow-up are aligned, thereby avoiding the immortal time bias that arises when treatment duration is defined post hoc (17). Nevertheless, important limitations remain. Residual confounding cannot be fully excluded, particularly for clinical factors influencing treatment decisions over time that may be incompletely captured in retrospective data. Given our small sample size, it was not possible to build weighting models allowing a perfect covariate balance, and weight variability may have reduced precision even after truncation. Another limitation is the relatively small number of patients in each group included in the emulated trials. The absence of a difference in mortality between patients treated with short and long courses of L-AmB could be due to a lack of power, as reflected in the large confidence interval of the calculated HR. Statistical power depends on the ability to achieve adequate covariate balance between groups and on the distribution of weights, which cannot be known in advance; therefore, an *a priori* calculation is not possible. Although non-inferiority margins could have been defined post hoc, doing so would have been methodologically unsound and potentially misleading. However, these results could help build a trial comparing different therapeutic strategies in PM. Indeed, the recent availability of new rapid diagnosis tools (including Mucorales qPCR) is a step toward the possibility of clinical trials.

Our results may lead to the consideration of using a triazole as first-line therapy in patients at risk of experiencing L-AmB toxicity. Indeed, some patients are already treated with triazole as first-line therapy (18). However, there is no clinical trial comparing L-AmB with posaconazole or isavuconazole as first-line therapy for the treatment of mucormycosis. The VITAL single-arm trial evaluated the efficacy of isavuconazole using a matched case-control analysis with a historical cohort of patients treated with L-AmB (6). The MoveOn study evaluating posaconazole efficacy compared five patients receiving posaconazole with 15 matched patients treated with L-AmB from the Fungiscope registry (7). Their results support comparable efficacy of L-AmB and triazole as first-line therapy. However, these studies suffered from the typical limitations of studies on rare diseases, including limited size and a non-randomized design. Moreover, several observations in our cohort are not in favor of using triazole as first-line therapy: 14 patients developed PM while receiving posaconazole prophylaxis; progression of mucormycosis was observed in 10 patients initially treated with L-AmB and then switched to a triazole; and a favorable outcome of mucormycosis was observed in half of the patients switched back to L-AmB. These observations argue for a better efficacy of L-AmB over triazole as first-line treatment.

Our study did not find improved survival in patients receiving combination therapy; however, this result is subject to the same limitations discussed above. While both posaconazole and isavuconazole combined with L-AmB have shown a synergic effect *in vitro* and in murine models (9, 10, 19), clinical evidence is lacking. A large retrospective study including 1,733 patients with COVID-associated mucormycosis found a better survival in patients receiving a primary combination therapy in multivariate analysis. However, results are inconclusive regarding severely immunosuppressed patients. In the study by Kyvernitakis et al., which included 106 HM patients, initial combination therapy had no impact on mortality after propensity score adjustment (20). However, half of the combination regimen consisted of posaconazole and L-AmB, while the other half consisted of an echinocandin and L-AmB. Echinocandins are now known for exhibiting limited activity against Mucorales compared to other agents. In both the study by Kyvernitakis and ours, the effect of isavuconazole and L-AmB combination therapy was not investigated. Studies evaluating the effect of such a combination would be of great interest.

We observed a high 180-day mortality rate of 54%. The prognosis of mucormycosis has been correlated with time to treatment initiation (21, 22). In our cohort, a treatment active against Mucorales was started a median of 11 days after the first symptom. Of note, receiving active empiric antifungal therapy was associated with worse outcomes in the multivariate Cox model; however, the association did not reach significance, presumably reflecting the severity of these patients. Very few patients (16%) received an empiric antifungal therapy that was initially active against Mucorales, while most received empiric antifungal therapy inactive against Mucorales (41%). This observation questions the role and nature of empirical therapy in patients with a high suspicion of invasive fungal disease. Clinicians often made the choice to initiate an empiric therapy of voriconazole, which is recommended as first-line therapy in pulmonary aspergillosis (23, 24). Mucormycosis shares clinico-radiological similarities with invasive pulmonary aspergillosis, although some radiological features are predictive of PM (25, 26). Invasive aspergillosis is five times more frequent than mucormycosis in France (27). However, the incidence of mucormycosis is rising, partly due to a wider use of PCR-based methods for diagnoses (3, 28). In the prospective ModiMucor study, which included 232 cases with suspected invasive mold disease, 29% of patients were diagnosed with IPA and 12% with PM (29). Patients with a high suspicion of invasive mold infection may benefit from an empirical therapy active against both Mucorales and *Aspergillus*. Moreover, given the high rates of Mucorales and *Aspergillus* co-infection (29–31), this empirical therapy may be maintained until all mycological evidence is available and co-infection is ruled out.

In summary, emulated trials found that a 14-day course of L-AmB followed by triazole therapy was as effective as longer courses while potentially reducing L-AmB side effects, whereas combination therapy did not improve prognosis. These findings must be interpreted with caution, given the retrospective design and the small sample size inherent to the rarity of the disease, despite extensive efforts to minimize bias. Nevertheless, our results could inform the design of randomized clinical trials evaluating first-line therapeutic strategies for pulmonary mucormycosis.

## Data Availability

The data supporting the findings of this study consist of confidential clinical data and cannot be made publicly available due to ethical and legal restrictions. De-identified data may be made available upon reasonable request to the corresponding authors. All of the individual participant data collected during this research will be available one year following publication, with no end date, for researchers who provide a methodologically sound proposal to achieve aims in the approved proposal, after approval of a proposal from the corresponding authors. Proposals should be directed at anne.coste@chu-brest.fr. To gain access, data requestors will need to sign a data access agreement.

## SUPPLEMENTAL MATERIAL

Supplemental material aac.01523-25-s0001.docx Methods; Tables S1 to S8; Fig. S1 to S3.

ASM does not own the copyrights to Supplemental Material that may be linked to, or accessed through, an article. The authors have granted ASM a non-exclusive, world-wide license to publish the Supplemental Material files. Please contact the corresponding author directly for reuse.
