# Supplementary material for: A step toward defining optimal liposomal amphotericin B treatment duration in pulmonary mucormycosis: results from emulated trials
Source: Antimicrob Agents Chemother. 2026 Apr 29;70(6):e01523-25. doi: 10.1128/aac.01523-25 (PMC13231879; doi:10.1128/aac.01523-25)
Supplement: Supplemental material — Methods; Tables S1 to S8; Fig. S1 to S3. [file aac.01523-25-s0001.docx]

**Supplementary data**

**Methods.**

Survival curves from diagnosis were estimated using Kaplan-Meier method. Since there was no censoring during the first 45 days of follow-up, 45-day mortality was analyzed using logistic regression. A multivariable model was fitted using a predefined set of predictors, without variable selection. For day 180 survival, Cox regression was used, with the same predictors, and a baseline hazard stratified according to surgery. The proportional hazards assumption was evaluated by examination of Schoenfeld residuals and Grambsch-Therneau lack-of-fit test. In case of violation, time-dependent effects were added to the model.

Two different causal analyses were undertaken to estimate the effect of liposomal amphotericin B (L-AmB) treatment duration and surgery, while adjusting for immortal time bias and indication bias. We first emulated a trial comparing L-AmB for 28 days or less to L-AmB for more than 28 days using a cloning and censoring approach.^1,2^ The approach for emulation of a target trial using observational data we used required the following steps: (1) specification of the target trial and eligibility criteria; (2) cloning participants so that each study participant is allocated to each treatment duration group; (3) censoring the clones when their actual treatment deviates from their group (e.g. for a clone in the ‘> 28 days’ group stops treatment at day 18); (4) derive inverse probability weights to account for the selection bias due to artificial censoring; (5) analyzing the data using those weights. We considered a hypothetical target trial were participants would ideally be randomized between receiving L-AmB for at most 28 days or more than 28 days. Eligible patients would be those for whom physicians would consider prescribing L-AmB for pulmonary mucormycosis within 10 days of the diagnosis. The primary outcome would be survival up to 180 days of randomization, and the between-group comparison (estimands) would be expressed in terms of difference in 45 days survival, difference in 180 days survival, difference in 180-day restricted mean survival time and hazard ratio after 28 days (no difference should be expected before day 28). In the emulated trial, the same target population, intervention and outcomes were used, but follow-up started from actual treatment initiation instead of randomization (Table S1). To adjust for indication bias in the actual decisions to stop or continue treatment, a prognostic score was estimated using a Cox model and time-fixed effects only, and used as a covariate in a Cox model for the weights (the follow-up time and event being then time to deviation from assigned strategy and such a deviation, respectively), with additional adjustment for response to treatment (progression vs. stable disease or response) as a time-dependent variable. In the ‘up to 28 days’ group, since artificial censoring could only occur at 28 days, a logistic regression model was used to compute the probability of remaining uncensored and weights.^2^ Weights corresponded to the inverse of the group-specific probability of remaining (artificially) uncensored during follow-up, given individual baseline risk and response to treatment. To ensure stability, weights were truncated at their 99^th^ percentile. The final analysis consisted in estimating the survival in each group in the weighted sample,^3^ and computing the differences at prespecified timepoints, as well as the 180-day restricted mean survival difference.^4^ Last, the hazard ratio between groups after day 28 was estimated in a weighted Cox model. Owing to the complexity of trial emulation, all standard errors of estimates were obtained using bootstrapping, where all steps of trial emulation were repeated in 999 bootstrap samples.

A similar approach (in particular cloning, censoring, and weighting) was used to emulate a trial in which participants still under L-AmB treatment after 14 days would be randomized between stopping or continuing treatment. To accommodate the possibility that eligible individuals would not stop treatment exactly at day 14, but possibly in the next few days, a 7 days grace period was used.

Last, we also emulated a trial comparing combination L-AmB therapy to any other treatment. Since both were determined at time of the first treatment give and with an analysis according to the first treatment, irrespective of later changes, there were no time-dependent issues, contrary to the stopping at day 14 or more than 28 days vs less emulated trials. To balance groups at baseline, we inverse probability of treatment weighting.^5^ The propensity score was estimated using logistic regression with the following predefined variables: age at diagnosis, sex, diagnosis in the ICU, dyspnea at diagnosis, neutropenia < 500/mm^3^ in the month preceding diagnosis, disseminated disease, treatment before formal diagnosis and ground-glass opacity on the CT-scan performed closest to diagnosis. We then aimed at achieving the best balance of weighted groups, as measured by standardized mean differences (SMDs).^6^ A SMD less than or equal to 0.15 was considered as indicating successful balance. Groups were compared in terms of survival difference, 180-day RMST and hazard ratio in the weighted sample. Weighted robust standard error were used for survival values and differences and the hazard ratio.{Citation}

All analyses were carried out using the R statistical software version 4.4.0 (R Foundation for Statistical Computing. Vienna, Austria, 2021).

Bibliography

1 Hernán MA. How to estimate the effect of treatment duration on survival outcomes using observational data. *BMJ* 2018; **360**: k182.

2 Maringe C, Benitez Majano S, Exarchakou A, *et al.* Reflection on modern methods: trial emulation in the presence of immortal-time bias. Assessing the benefit of major surgery for elderly lung cancer patients using observational data. *Int J Epidemiol* 2020; **49**: 1719–29.

3 Cole SR, Hernán MA. Adjusted survival curves with inverse probability weights. *Comput Methods Programs Biomed* 2004; **75**: 45–9.

4 Royston P, Parmar MKB. The use of restricted mean survival time to estimate the treatment effect in randomized clinical trials when the proportional hazards assumption is in doubt. *Stat Med* 2011; **30**: 2409–21.

5 Lunceford JK, Davidian M. Stratification and weighting via the propensity score in estimation of causal treatment effects: a comparative study. *Stat Med* 2004; **23**: 2937–60.

6 Franklin JM, Rassen JA, Ackermann D, Bartels DB, Schneeweiss S. Metrics for covariate balance in cohort studies of causal effects. *Stat Med* 2014; **33**: 1685–99.

7 Cafri G, Austin PC. Variance estimation of the risk difference when using propensity-score matching and weighting with time-to-event outcomes. *Pharm Stat* 2023; **22**: 880–902.

**Table S1. Specification and emulation of a target trial of L-AmB discontinuation at 14 days.**

| **Component** |  | **Target trial** |  | **Emulated trial using observational data** |
| --- | --- | --- | --- | --- |
| **Design** |  | Multicenter open-label two-parallel arm superiority randomized trial. |  |  |
| **Objective** |  | Compare treatment by L-AmB beyond 14 days versus stopping treatment at 14 days |  | Same |
| **Eligibility criteria** |  | Patients diagnosed with pulmonary mucormycosis (modified EORTC criteria) for whom L-AmB treatment is decided within 10 days of diagnosis, and who are still alive and under treatment at 14 days |  | Same |
| **Exclusions** |  | Patients deceased or switched to another treatment before 14 days |  | Same |
| **Treatment strategies** |  | 1. Stop treatment at 14 days 2. Continue treatment after 14 days |  | Same |
| **Treatment assignment** |  | Patients are randomly assigned to either strategy |  | Patients are non-randomly assigned to a treatment strategy. Randomization is emulated via cloning of patients in both groups. A grace period of 7 days is used. |
| **Outcome** |  | Death from all causes within 180 days |  | Same |
| **Type of outcome** |  | Failure time |  | Same |
| **Follow up** |  | Follow up starts at 14 days of treatment |  | Follow up starts at 14 days of treatment |
| **Censoring** |  | Loss to follow up, administrative censoring |  | Loss to follow up, administrative censoring |
| **Adjustment variables** |  | Age, sex, diagnosis in the ICU, dyspnea at diagnosis, neutropenia before or at diagnosis, disseminated bronchopulmonary disease, pleural effusion on CT-scan, ground-glass opacity on CT-scan |  | Same |
| **Causal contrast** |  | *Per protocol* |  | *Per protocol*: we do not know what the intention to treat was from the data; In each group of the emulated trial, patients who deviate from the protocol are censored at their time of deviation |
| **Estimands** |  | Differences between groups in 45-day and 180-day survival, and restricted mean survival time at 180 days, and hazard ratio after 28 days |  | Same |

**Table S2. Specification and emulation of a target trial of L-AmB treatment duration of 28 days or less versus more than 28 days.**

| **Component** |  | **Target trial** |  | **Emulated trial using observational data** |
| --- | --- | --- | --- | --- |
| **Design** |  | Multicenter open-label two-parallel arm superiority randomized trial. |  |  |
| **Objective** |  | Compare treatment by L-AmB for more than 28 days versus 28 days or less |  | Same |
| **Eligibility criteria** |  | Patients diagnosed with pulmonary mucormycosis (modified EORTC criteria) for whom L-AmB treatment is decided within 10 days of diagnosis |  | Same |
| **Exclusions** |  | Contraindication to L-AmB |  | Same |
| **Treatment strategies** |  | 1. Treatment for at most 28 days 2. Treatment for more than 28 days |  | Same |
| **Treatment assignment** |  | Patients are randomly assigned to either strategy |  | Patients are non-randomly assigned to a treatment strategy. Randomization is emulated via cloning of patients in both groups. |
| **Outcome** |  | Death from all causes within 180 days |  | Same |
| **Type of outcome** |  | Failure time |  | Same |
| **Follow up** |  | Follow up starts at treatment assignment |  | Follow up starts at treatment initiation |
| **Censoring** |  | Loss to follow up, administrative censoring |  | Loss to follow up, administrative censoring |
| **Adjustment variables** |  | Age, sex, diagnosis in the ICU, dyspnea at diagnosis, neutropenia before or at diagnosis, disseminated bronchopulmonary disease, pleural effusion on CT-scan, ground-glass opacity on CT-scan |  | Same |
| **Causal contrast** |  | *Per protocol* |  | *Per protocol*: we do not know what the intention to treat was from the data; In each group of the emulated trial, patients who deviate from the protocol are censored at their time of deviation |
| **Estimands** |  | Differences between groups in 45-day and 180-day survival, and restricted mean survival time at 180 days, and hazard ratio after 28 days |  | Same |

**Table S3. Balance diagnostics for the 14-day L-AmB treatment discontinuation emulated trial just after the 7-day grace period. Only individuals included in the emulated trial and still alive at 21 days from treatment initiation (7 days after the 14-day timepoint) are included in the table.**

|  | **Before weighting** | | | **After weighting** | | |
| --- | --- | --- | --- | --- | --- | --- |
|  | **Continue L-AmB** | **Stop L-AmB** |  | **Continue L-AmB** | **Stop L-AmB** |  |
|  | **N = 45** | **N = 11** | **SMD** | **N = 58.0*** | **N = 55.8*** | **SMD** |
| Age, mean (SD) y | 51.6 (17.5) | 47.2 (16.0) | 0.262 | 52.3 (17.2) | 45.2 (13.7) | 0.452 |
| Male, no. (%) | 36 (80.0) | 7 (63.6) | 0.370 | 44.7 (77.1) | 41.8 (74.9) | 0.051 |
| Diagnosis in the ICU, no. (%) | 14 (31.1) | 3 (27.3) | 0.085 | 20.1 (34.6) | 17.3 (31.1) | 0.076 |
| Dyspnea at diagnosis, no. (%) | 22 (48.9) | 6 (54.5) | 0.113 | 29.5 (50.9) | 30.9 (55.4) | 0.091 |
| Neutropenia before or at diagnosis, no. (%) | 26 (57.8) | 7 (63.6) | 0.120 | 32.8 (56.6) | 33.2 (59.6) | 0.060 |
| Treatment started ≥ 2 days before diagnosis, no. (%) | 17 (37.8) | 1 (9.1) | 0.720 | 20.8 (35.9) | 12.3 (22.0) | 0.310 |
| Disseminated bronchopulmonary disease, no. (%) | 10 (22.2) | 4 (36.4) | 0.315 | 13.7 (23.6) | 22.2 (39.7) | 0.351 |
| Pleural effusion on CT-scan, no. (%) | 19 (42.2) | 5 (45.5) | 0.065 | 26.0 (44.9) | 25.1 (45.1) | 0.003 |
| Ground-glass opacity on CT-scan, no. (%) | 29 (64.4) | 6 (54.5) | 0.203 | 38.4 (66.2) | 30.3 (54.3) | 0.246 |
| 180-day mortality score, mean (SD)^†^ | 1.5 (0.9) | 1.7 (0.8) | 0.281 | 1.6 (0.9) | 1.7 (0.7) | 0.185 |
| Response to treatment at day 14, no. (%) | 19 (42.2) | 8 (72.7) | 0.649 | 27.4 (47.2) | 26.0 (46.6) | 0.013 |

SMD: standardized mean difference; SD: standard deviation.

* Sum of weights

† Linear predictor of a Cox model with age at diagnosis, sex, diagnosis in the ICU, dyspnea at diagnosis, neutropenia before or at diagnosis, disseminated bronchopulmonary disease, pleural effusion on CT-scan, and ground-glass opacity on CT-scan with time-fixed effects.

**Table S4. Comparison of survival according to stopping or continuing L-AmB treatment after 14 days**

| **Estimand** | **Stop treatment** | **Continue treatment** | **Difference (95% CI)** |
| --- | --- | --- | --- |
| 45-day survival | 70% (31 to 94) | 69% (54 to 82) | -2% (-30 to 36) |
| 180-day survival | 58% (25 to 87) | 52% (37 to 66) | -7% (-34 to 26) |
| 180-day RMST (days) | 112 (60 to 147) | 101 (80 to 120) | -11 (-46 to 40) |
| Hazard ratio | — | — | 0.80 (0.29 to 1.99) |

RMST: restricted mean survival time

**Table S5. Balance diagnostics for the ≤ 28 days vs > 28 days L-AmB treatment duration emulated trial.** Only individuals included in the emulated trial and still alive at 28 days from treatment initiation are included in the table.

|  | **Before weighting** | | | | **After weighting** | | | |
| --- | --- | --- | --- | --- | --- | --- | --- | --- |
|  | **L-AmB duration > 28 days** | **L-AmB duration ≤ 28 days** |  | **L-AmB duration > 28 days** | | **L-AmB duration ≤ 28 days** |  |  |
|  | **N = 39** | **N = 18** | **SMD** | **N = 62.5*** | | **N = 57.0*** | **SMD** |  |
| Age, mean (SD) y | 50.9 (16.2) | 51.8 (20.3) | 0.047 | 51.9 (16.2) | | 54.0 (20.0) | 0.114 |  |
| Male, no. (%) | 31 (79.5) | 12 (66.7) | 0.292 | 49.5 (79.2) | | 38.4 (67.4) | 0.271 |  |
| Diagnosis in the ICU, no. (%) | 11 (28.2) | 3 (16.7) | 0.279 | 18.4 (29.5) | | 6.5 (11.4) | 0.460 |  |
| Dyspnea at diagnosis, no. (%) | 17 (43.6) | 10 (55.6) | 0.241 | 26.8 (42.8) | | 27.4 (48.0) | 0.105 |  |
| Neutropenia before or at diagnosis, no. (%) | 23 (59.0) | 9 (50.0) | 0.181 | 34.6 (55.4) | | 30.9 (54.2) | 0.024 |  |
| Treatment started ≥ 2 days before diagnosis, no. (%) | 16 (41.0) | 1 (5.6) | 0.924 | 23.0 (36.8) | | 8.0 (14.0) | 0.542 |  |
| Disseminated bronchopulmonary disease, no. (%) | 10 (25.6) | 4 (22.2) | 0.080 | 15.9 (25.4) | | 20.0 (35.0) | 0.211 |  |
| Pleural effusion on CT-scan, no. (%) | 16 (41.0) | 6 (33.3) | 0.160 | 25.8 (41.2) | | 18.5 (32.5) | 0.182 |  |
| Ground-glass opacity on CT-scan, no. (%) | 27 (69.2) | 9 (50.0) | 0.400 | 41.8 (66.9) | | 37.2 (65.2) | 0.036 |  |
| 180-day mortality score, mean (SD)^†^ | 1.5 (0.9) | 1.3 (1.0) | 0.202 | 1.4 (0.9) | | 1.4 (1.1) | 0.055 |  |
| Response to treatment at day 14, no. (%) | 18 (46.2) | 15 (83.3) | 0.845 | 37.3 (59.7) | | 33.0 (57.8) | 0.038 |  |

SMD: standardized mean difference; SD: standard deviation.

* Sum of weights

† Linear predictor of a Cox model with age at diagnosis, sex, diagnosis in the ICU, dyspnea at diagnosis, neutropenia before or at diagnosis, disseminated bronchopulmonary disease, pleural effusion on CT-scan, and ground-glass opacity on CT-scan with time-fixed effects.

**Table S6: Comparison of survival according to L-AmB treatment duration in the 28-day emulated trial.**

| **Estimand** | **28 days or less** | **More than 28 days** | **Difference (95% CI)** |
| --- | --- | --- | --- |
| 45-day survival | 58% (30 to 73) | 59% (47 to 71) | 1% (-13 to 29) |
| 180-day survival | 41% (24 to 63) | 47% (34 to 59) | 5% (-16 to 24) |
| 180-day RMST (days) | 100 (70 to 125) | 101 (82 to 119) | 1 (-21 to 30) |
| Hazard ratio after day 28 | — | — | 0.98 (0.43 to 5.02) |

RMST: restricted mean survival time

**Table S7: Balance diagnostics before and after weighting in the combination L-AmB therapy emulated trial.**

|  | **Before weighting** | | | **After weighting** | | |
| --- | --- | --- | --- | --- | --- | --- |
|  | **Single therapy** | **Combination therapy** |  | **Single therapy** | **Combination therapy** |  |
|  | **N = 75** | **N = 18** | **SMD** | **N = 93.1*** | **N = 89.6*** | **SMD** |
| Age, mean (SD) y | 52.0 (17.9) | 50.4 (18.8) | 0.086 | 51.7 (18.1) | 52.5 (16.8) | 0.044 |
| Male, no. (%) | 53 (70.7) | 12 (66.7) | 0.086 | 65.2 (70.0) | 66.4 (74.2) | 0.094 |
| Diagnosis before treatment, no. (%) | 29 (38.7) | 3 (16.7) | 0.507 | 31.9 (34.2) | 24.5 (27.4) | 0.149 |
| Diagnosis in the ICU, no. (%) | 26 (34.7) | 8 (44.4) | 0.201 | 33.6 (36.1) | 29.6 (33.1) | 0.063 |
| Dyspnea at diagnosis, no. (%) | 39 (52.0) | 10 (55.6) | 0.071 | 49.6 (53.2) | 51.8 (57.9) | 0.093 |
| Neutropenia before or at diagnosis, no. (%) | 46 (61.3) | 11 (61.1) | 0.005 | 58.3 (62.6) | 56.8 (63.4) | 0.016 |
| Disseminated bronchopulmonary disease, no. (%) | 25 (33.3) | 10 (55.6) | 0.459 | 35.2 (37.8) | 32.5 (36.3) | 0.030 |
| Pleural effusion on CT-scan, no. (%) | 38 (50.7) | 11 (61.1) | 0.212 | 47.3 (50.8) | 41.5 (46.4) | 0.089 |
| Ground-glass opacity on CT-scan, no. (%) | 45 (60.0) | 12 (66.7) | 0.139 | 57.0 (61.2) | 53.3 (59.5) | 0.036 |
| 180-day mortality score, mean (SD)^†^ | 1.7 (1.1) | 2.2 (1.5) | 0.338 | 1.8 (1.1) | 1.8 (1.5) | 0.032 |

* Sum of weights

† Linear predictor of a Cox model with age at diagnosis, sex, diagnosis in the ICU, dyspnea at diagnosis, neutropenia before or at diagnosis, disseminated bronchopulmonary disease, pleural effusion on CT-scan, and ground-glass opacity on CT-scan with time-fixed effects.

**Table S8: Comparison of survival according to combination L-AmB therapy.**

| **Estimand** | **Single therapy** | **Combination therapy** | **Difference (95% CI)** |
| --- | --- | --- | --- |
| 45-day survival | 58% (48 to 71) | 42% (22 to 79) | -16% (-45 to 13) |
| 180-day survival | 43% (33 to 55) | 36% (17 to 74) | -7% (-35 to 22) |
| 180-day RMST (days) | 96 (80 to 111) | 85 (69 to 100) | -11 (-33 to 11) |
| Hazard ratio | — | — | 1.14 (0.56 to 2.32) |

RMST: restricted mean survival time

**Figure S1. Survival according to main risk factor**

Alt text: survival curve according to main risk factor, showing improved survival in patients with solid organ transplantation compared to hematological patients.

**Figure S2. Cumulative incidence of death according to cause of death**

Alt text: chart depicting cumulative incidence of death over time according to cause of death, showing higher incidence of death from mucormycosis compared to death from other causes, especially in the first 28 days.

**Figure S3. Overall survival up to day 180 according to L-AmB treatment duration in the 28-day emulated trial.**

Alt text: survival curve according to L-AmB treatment duration (> 28 days or ≤ 28 days) in the emulated trial, showing no difference between groups.
